# Supplementary figures and images for: Shifts in dominance of benthic communities along a gradient of water temperature and turbidity in tropical coastal ecosystems
Source: PeerJ. 2024 Apr 22;12:e17132. doi: 10.7717/peerj.17132 (PMC11044884; doi:10.7717/peerj.17132)

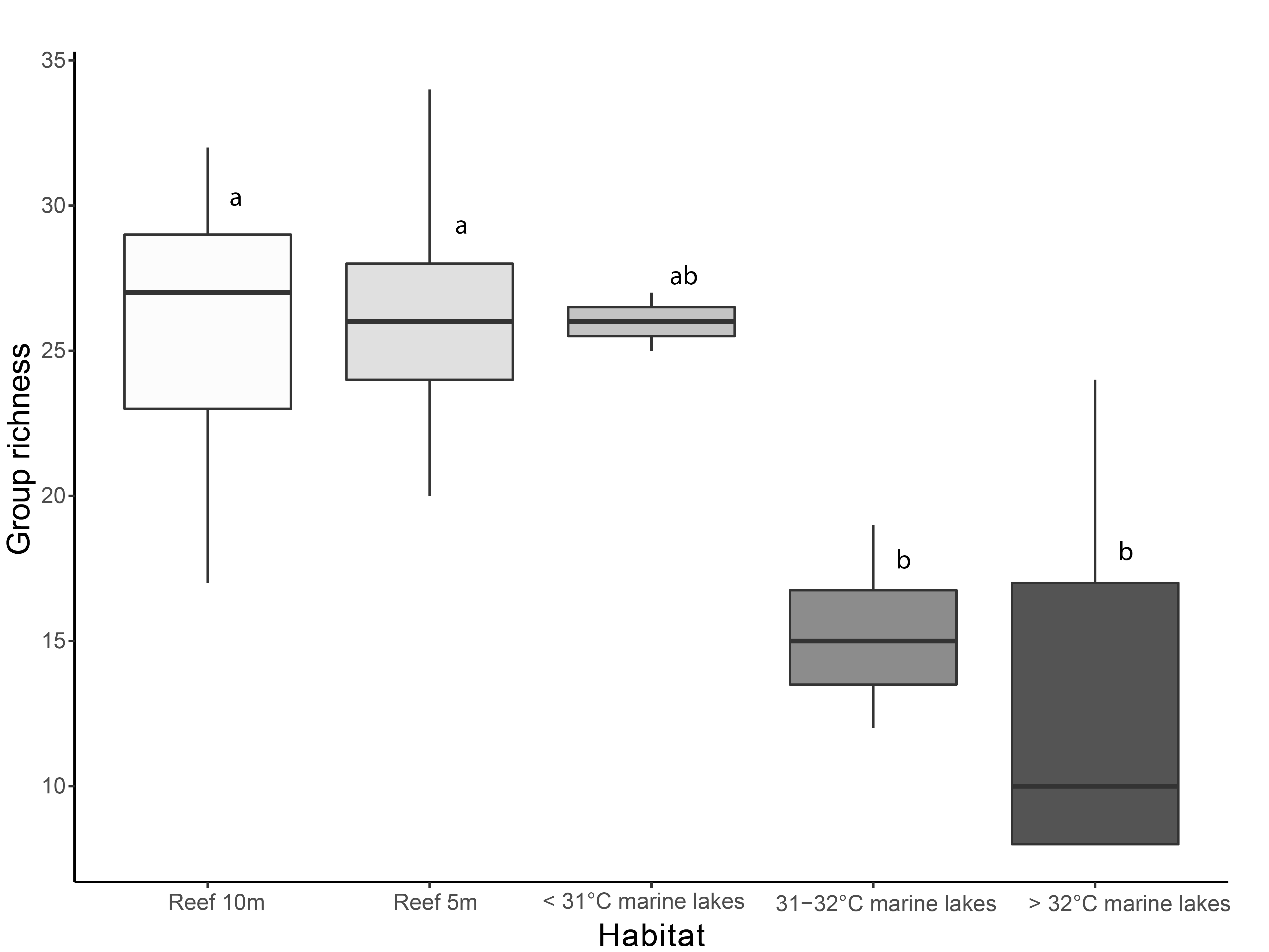

Supplement: Supplemental Information 1 — Different letters indicate significant differences (Kruskal–Wallis chi-squared = 24.541, df = 4, p < 0.001). [file peerj-12-17132-s001.jpg]

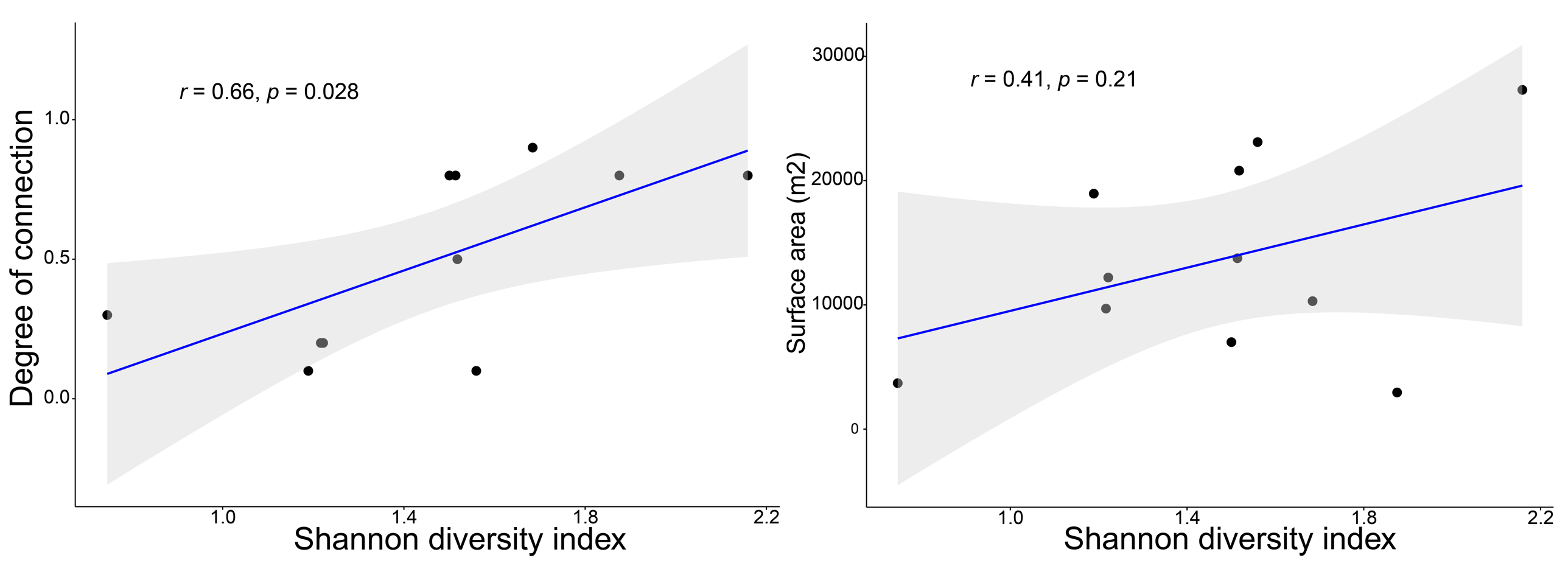

Supplement: Supplemental Information 2 [file peerj-12-17132-s002.jpg]

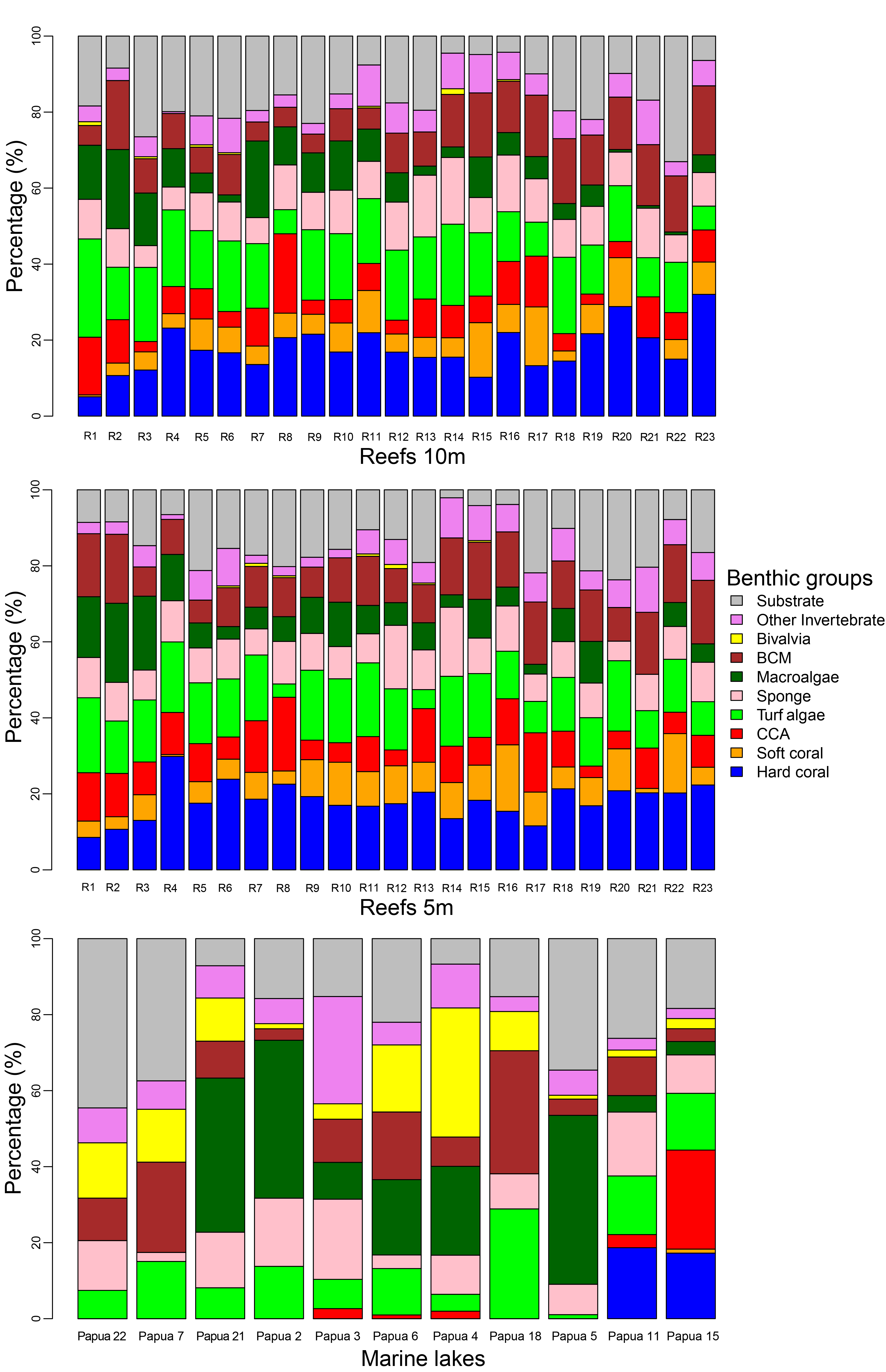

Supplement: Supplemental Information 3 — Note: CCA, Crustose Coralline Algae; BCM, Benthic Cyanobacterial Mats. [file peerj-12-17132-s003.jpg]

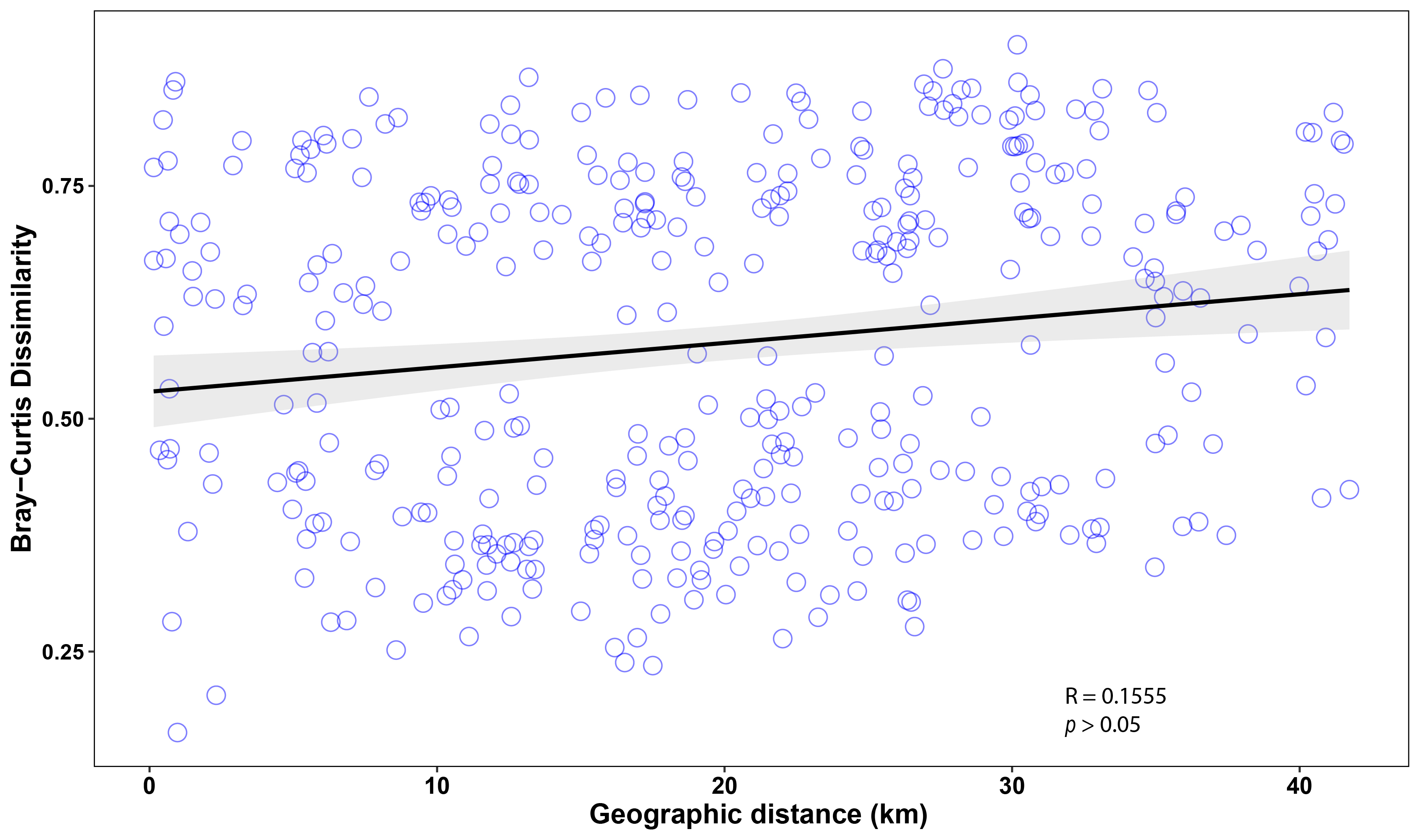

Supplement: Supplemental Information 4 — The closest distances between habitats appear more similar in community assemblages. [file peerj-12-17132-s004.jpg]
