# Supplementary material for: Shifts in dominance of benthic communities along a gradient of water temperature and turbidity in tropical coastal ecosystems
Source: PeerJ. 2024 Apr 22;12:e17132. doi: 10.7717/peerj.17132 (PMC11044884; doi:10.7717/peerj.17132)
Supplement: Supplemental Information 5 — Note: Millepora, Tubipora and Heliopora are classified into Hard Coral because they are calcareous reef framework builders. [file peerj-12-17132-s005.docx]

**Tabel S1.** The category and of major (course functional group) and finer-scale benthic groups that are used for the analysis.

| **Major benthic group** | **Finer-scale benthic group** |
| --- | --- |
| Hard Coral | Branching *Acropora* (AC_Br) |
|  | Digitate *Acropora* (AC_Di) |
|  | Encrusting *Acropora* (AC_En) |
|  | Submassive *Acropora* (AC_Sm) |
|  | Tabulate *Acropora* (AC_Tb) |
|  | Branching coral (CO_Br) |
|  | Encrusting coral (CO_Fo) |
|  | Foliose coral (CO_En) |
|  | Massive coral (CO_Ma) |
|  | Submassive coral (CO_Sm) |
|  | Mushroom coral (CO_Mu) |
|  | *Millepora* (CO_Me) |
|  | *Tubipora* (CO_Tu)  *Heliopora* (CO_Hl) |
| Soft Coral | Gorgonian (SC_Go) |
|  | Xenia (SC_Xe) |
|  | Other softcoral (SC_OT) |
| Crustose Coralline Algae (CCA) | Crustose Coralline Algae (CCA) |
| Turfalgae | Turfalgae (AL_TA) |
|  | Dead coral algae (AL_DC) |
| Sponge | Ball sponge (SP_Ba) |
|  | Branching sponge (SP_Br) |
|  | Digitate sponge (SP_Di) |
|  | Encrusting sponge (SP_En) |
|  | Excavating sponge (SP_Ex) |
|  | Massive sponge (SP_Ma) |
|  | Tube Sponge (SP_Tu) |
|  | Other sponge (SP_OT) |
| Macroalgae | *Caulerpa* (MA_Ca) |
|  | *Cladophora* (MA_Cl) |
|  | *Halimeda* (MA_Ha) |
|  | *Padina* (MA_Pa) |
|  | *Sargassum* (MA_Sa) |
|  | Other macroalgae (MA_OT) |
| Benthic Cyanobacterial Mats (BCMs) | *Lyngbya* (CY_Ly) |
|  | Red-brown mats (CY_RB) |
|  | Other BCM (CY_OT) |
| Bivalvia | Clam (BI_Cl) |
|  | Cockle (BI_Co) |
|  | Mussel (BI_Mu) |
|  | Oyster (BI_Oy) |
| Other invertebrates | Gastropoda Cerithidae (GA_Ce) |
|  | Gastropoda Muricidae (GA_Mr) |
|  | Gastropoda Neritidae (GA_Ne) |
|  | Gastropoda Potamididae (GA_Po) |
|  | Gastropoda Trochidae (GA_Tr) |
|  | Gastropoda other (GA_OT) |
|  | Ascidian colonial (OT_AC) |
|  | Ascidian solitary (OT_AS) |
|  | Echinoderm Asteroidea (OT_EA) |
|  | Echinoderm Crinoidea (OT_EC) |
|  | Echinoderm Echinoidea (OT_EE) |
|  | Echinoderm Holothuroidea (OT_EH) |
|  | Echinoderm Ophiuroidea (OT_EO) |
|  | Hydrozoa (OT_HY) |
|  | Polychaeta (OT_PO) |
|  | Zoanthid (OT_ZO) |
|  | Other biota (OT) |
| Substrates | Branch / Small-wood (SU_Br) |
|  | Dead shell (SU_Ds) |
|  | Leaf / Litter (SU_Le) |
|  | Mangrove root (SU_Mr) |
|  | Mud / Silt (SU_Mu) |
|  | Rubble (SU_Ru) |
|  | Rock (SU_Ro) |
|  | Sand (SU_Sa) |

Note: *Millepora, Tubipora* and *Heliopora* are classified into Hard Coral because they are calcareous reef framework builders.
