# Supplementary material for: Shifts in dominance of benthic communities along a gradient of water temperature and turbidity in tropical coastal ecosystems
Source: PeerJ. 2024 Apr 22;12:e17132. doi: 10.7717/peerj.17132 (PMC11044884; doi:10.7717/peerj.17132)
Supplement: Supplemental Information 6 [file peerj-12-17132-s006.docx]

**Table S2.** The percent cover of major benthic groups (mean ± standard deviation) from five habitat categories in Raja Ampat, Southwest Papua, Indonesia.

| **Benthic cover** | **Reefs 10m** | **Reefs 5m** | **<31°C lakes** | **31-32°C lakes** | **>32°C lakes** |
| --- | --- | --- | --- | --- | --- |
| Hard Coral | 17.6 ± 6.1 | 18.1 ± 4.7 | 18.0 ± 1 | 0 | 0 |
| Soft Coral | 6.6 ± 3.9 | 7.6 ± 4.1 | 0.5 ± 0.7 | 0 | 0 |
| Crustose Coralline Algae | 8.2 ± 4.4 | 9.4 ± 4.1 | 14.8 ± 16 | 0.7 ± 0.9 | 0.5 ± 1.2 |
| Turfalgae | 15.7 ± 4.8 | 14.4 ± 4.5 | 15.2 ± 0.3 | 11.6 ± 12.4 | 10.4 ± 3.7 |
| Sponge | 10.5 ± 3 | 10 ± 2.8 | 13.5 ± 4.7 | 7.8 ± 3 | 13.8 ± 7.1 |
| Macroalgae | 7.8 ± 5.8 | 8 ± 5.4 | 3.9 ± 0.6 | 21.9 ± 18.2 | 18.4 ± 21.1 |
| Benthic Cyanobacterial Mats | 11.3 ± 4.6 | 12.4 ± 3.5 | 6.8 ± 4.8 | 15.5 ± 12.6 | 11.8 ± 7.5 |
| Bivalvia | 0.2 ± 0.4 | 0.2 ± 0.3 | 2.2 ± 0.6 | 15.7 ± 13.9 | 9 ± 6 |
| Other Invertebrates | 6.1 ± 2.9 | 6.1 ± 3 | 2.9 ± 0.3 | 7 ± 3.2 | 12 ± 9.1 |
| Substrate | 15.9 ± 7.7 | 13.9 ± 6.5 | 22.3 ± 5.6 | 19.6 ± 11.8 | 24 ± 16.1 |
